# Functioning and quality of life among treatment-engaged adults with psychotic disorders in urban Tanzania: Baseline results from the KUPAA clinical trial

**DOI:** 10.1371/journal.pone.0304367

**Published:** 2024-06-18

**Authors:** Joseph R. Egger, Sylvia Kaaya, Praxeda Swai, Paul Lawala, Liness Ndelwa, Joseph Temu, Eliasa Swata Bukuku, Ellen Lukens, Ezra Susser, Lisa Dixon, Anna Minja, Rosarito Clari, Alyssa Martinez, Jennifer Headley, Joy Noel Baumgartner

**Affiliations:** 1 Duke Global Health Institute, Duke University, Durham, NC, United States of America; 2 Muhimbili University of Health & Allied Sciences, Dar es Salaam, Tanzania; 3 Mbeya Zonal Referral Hospital, Mbeya, Tanzania; 4 School of Social Work, Columbia University, New York, NY, United States of America; 5 New York State Psychiatric Institute, New York, NY, United States of America; 6 Mailman School of Public Health, Columbia University, New York, NY, United States of America; 7 Department of Psychiatry, Columbia University, New York, NY, United States of America; 8 School of Social Work, University of North Carolina at Chapel Hill, Chapel Hill, NC, United States of America; University of Botswana, BOTSWANA

## Abstract

**Background:**

There is a treatment gap for those living with severe mental illnesses in low- and middle-income countries, yet not enough is known about those who are currently accessing clinical services. A better understanding of potentially modifiable factors associated with functioning and quality of life will help inform policies and programming.

**Aims:**

To describe the functioning and quality of life for a psychiatric treatment-engaged population living with psychotic disorders in two urban areas of Tanzania, and to explore their respective correlates.

**Methods:**

This study analyzed cross-sectional data from 66 individuals enrolled in the Kuwezeshana Kupata Uzima (KUPAA) pilot clinical trial who had a diagnosis of schizophrenia or schizoaffective disorder, recent relapse, and who were receiving outpatient treatment. Baseline functioning (WHO Disability Assessment Schedule 2.0) and quality of life (WHO Quality of Life BREF scale) were measured. Univariable and multivariable regression analyses were conducted to determine correlates of functioning and quality of life.

**Results:**

Adjusted analyses indicated that higher disability was associated with higher food insecurity, more symptomatology, more self-stigma, less instrumental support, less hope, lower self-efficacy, and/or lower levels of family functioning. Higher quality of life was associated with higher levels of self-efficacy, more hopefulness, more instrumental support, less self-stigma, and better family functioning.

**Conclusions:**

Identification of factors associated with disability and quality of life can help clinicians and policymakers, as well as consumers of mental health services, to better co-design and target psychosocial interventions to optimize their impact in low-resource settings.

**Trial registration:**

**Trial registration:** ClinicalTrials.gov # NCT04013932, July 10, 2019.

## Introduction

Psychotic disorders contribute substantially to the global burden of disease, including in low- and middle-income countries (LMICs). Global calls advocate for higher quality of care and more recovery-oriented psychiatric services that not only reduce symptoms, relapses, and hospitalizations, but also reduce disability and improve quality of life [1, 2, 3, 4]. Schizophrenia and other psychotic disorders can have a profound impact on both functioning and quality of life; evidence suggests that psychosocial interventions in tandem with pharmacological interventions can have a positive impact on these important non-clinical outcomes [5, 6, 7].

Several studies have described the level of functioning, quality of life and other characteristics of persons diagnosed with psychotic disorders in LMIC settings, such as Tanzania. Cross-sectional clinical studies of individuals diagnosed with psychotic disorders in China, Bangladesh and Ethiopia all found participants to have relatively low levels of quality of life using the World Health Organization Quality of Life—Brief version (WHOQOL-BREF) measure, particularly in the domains of psychological health and social relationships [8, 9, 10], which is consistent with previous studies in high-income country populations [11, 12]. A review examining psychiatric symptoms and quality of life among those with schizophrenia, largely in high-income countries, found higher positive and negative symptoms were significantly associated with worse quality of life (13). Regarding disability, a study of individuals with schizophrenia identified via clinics in Nigeria found that levels of disability are much higher compared with a random community-based sample and that disability was particularly high in the Participation in Society domain of the World Health Organization Disability Assessment Schedule-Second Version (WHODAS 2.0) [13]. Additional studies conducted in Nigeria reported that reduced quality of life and increased disability are also associated with increased symptom severity, as measured by the Positive and Negative Syndrome Scale (PANSS) [14, 15, 16].

Pharmacological treatments for psychotic disorders can be effective for managing symptoms but may not on their own result in associated improvements in functioning and quality of life. Thus, global guidelines recommend a combination of pharmacological and psychosocial interventions such as psychoeducation and social support [5, 17, 18, 19]. A 2021 systematic review of the frequency and correlates of severe mental illness found that high levels of self-stigma are often associated with poor clinical and functional outcomes, such as lower quality of life in low-resource settings [20, 21]. Relatedly, hopefulness and religiosity have been found to be associated with better quality of life [22, 23, 24, 25]. These findings suggest that individual and family-based psychosocial interventions that target improving or building upon social, emotional, spiritual and/or relational components may be beneficial in decreasing disability and improving quality of life in people living with a psychotic disorder.

In Tanzania, as in many other parts of East Africa, families are fundamental for mental health treatment—both culturally, in terms of shared decision-making for treatment options, and as the main source of support in resource-scarce environments [26]. The implementation and evaluation of culturally adapted, evidence-based psychosocial interventions aimed at addressing these factors, such as Family Psychoeducation [5, 19] could improve functioning and disability differentially by socio-demographic characteristics or via a variety of associated pathways that are under-explored such as hopefulness, family functioning, self-efficacy and instrumental support [27]. The present study focuses on the baseline data from the KUPAA pilot trial, which tested a culturally adapted form of family psychoeducation, (Clinicaltrials.gov ID # NCT04013932) to describe the level of disability and quality of life in a treatment-engaged Tanzanian patient population with psychotic disorders. The primary aim of the study was to explore the associations that disability and quality of life have with potentially modifiable factors at the individual or family level that psychosocial interventions, such as KUPAA, could target. We hypothesize that perceived level of support from family and caregivers, as well as one’s self-efficacy and hopefulness are positively associated with one’s quality of life and negatively associated with one’s level of disability. The development of cost-effective interventions that target modifiable protective factors, such as self-efficacy, may have the strongest effects on mediating improvements in disability and quality of life for those living with psychotic disorders in lower-resource environments like Tanzania, and globally.

## Methods

### Study design and participants

This current study uses cross-sectional, pre-intervention baseline data from an individually randomized group treatment (IRGT) trial in two tertiary level hospital facilities in Dar es Salaam and Mbeya, Tanzania. The parent study was designed as a two-arm, parallel IRGT trial; the primary objective of which is to estimate efficacy, and to explore potential mediators of the culturally tailored family psychoeducation intervention for individuals with psychotic disorders and their relatives in Tanzania. Main findings on the hypothesized outcomes of the IRGT trial (reduced disability and improved quality of life) will be published separately.

Statistical power was originally calculated for the KUPAA clinical trial and not for this baseline study. However, we estimate that our sample of 66 patient participants will provide 82% power to detect a correlation coefficient between two continuous variables (e.g., disability, self-efficacy) of 0.35 with an alpha level of 0.05 and assuming a null hypothesized correlation of 0.

### Study sites and participant recruitment

Baseline data collection occurred between September 3, 2019 and November 1, 2019 at each of the two clinic sites in Tanzania: Muhimbili National Hospital (MNH), affiliated with Muhimbili University of Health and Allied Sciences (MUHAS); and Mbeya Zonal Referral Hospital. MNH is the national referral and teaching hospital in Dar es Salaam, the commercial capital of Tanzania with a catchment area of approximately four million people. The Department of Psychiatry and Mental Health at MNH provides inpatient and outpatient care, and has a bed capacity of 70. Staff includes psychiatrists, psychiatric nurses, social workers, psychologists and occupational therapists. Outpatient services are offered daily and primarily focus on medication management although there are also ad hoc drop-in psychoeducation sessions for patients and relatives, and family meetings for patients who are experiencing frequent relapses.

Mbeya Zonal Referral Hospital (MZRH) is situated in Mbeya city, 900 km from Dar es Salaam and 100 kilometers from the Tanzania- Zambia border. MZRH is the only referral facility in the southern part of the country with a catchment area of approximately two million. The Psychiatry and Mental Health Unit has a 24-bed capacity, and outpatient clinics are held three times a week. Department staff include one psychiatrist, general practitioners, a clinical psychologist, psychiatric nurses, and social workers. Currently, staff conduct individual family conferences only for patients with frequent relapses and more than three admissions in a year. There are no regularly offered and standardized psychosocial services for clients with psychotic disorders at either hospital at the time of writing this report.

Adults attending one of the two study psychiatric outpatient clinics were eligible for inclusion if they were 18–50 years old, had a diagnosed psychotic disorder, and had a psychiatrically-related hospitalization or non-hospitalized psychiatric relapse within the past 12 months. A non-hospitalized relapse could include uncontrolled (but not eliminated) symptoms such as escalation of disorganized behavior and/or recurrence of symptoms that had previously been better controlled as per clinical judgement of our study psychiatrists (SK, PL, PS). All participants had ICD-10 diagnostic codes of either Schizophrenia [F20] (n = 56), or Schizoaffective disorder [F25] (n = 10). Comorbid diagnoses were acceptable for inclusion, (i.e., F12.15, ‘Cannabis abuse with psychotic disorder’) as long as they had a primary ICD-10 of F20, F21, F22 or F25. Because patient record keeping at psychiatric clinics is variable (e.g. daily ledger for tracking patients seen, medications dispensed, etc.) and some clinicians might have used a code number and/or the disorder name, diagnostic eligibility was confirmed for all by one of the three study team psychiatrists via further review of full patient notes/records and clinical interviews. Participants were ineligible if presence of co-morbid developmental disorder, dementia, or other severe cognitive deficit rendered the individual unable to provide informed consent.

Study brochures were posted at both study sites around the psychiatry departments and pharmacies. During enrollment, research assistants (RAs) were situated onsite at the clinics during busy outpatient clinic hours to identify potential participants and provide study information. If RAs had any concerns about capacity, psychiatrists at each study site were available to assess and determine capacity to consent to research participation. Following enrollment, individuals were randomized in equal allocation to the KUPAA study arm or usual care using Stata software. Randomization was stratified by study site, patient-participant sex and length of illness to better balance study arms.

### Data collection

After recruitment, baseline interviews were conducted with study participants. Interviewers collected data on participant socio-demographics, a range of psychometric instruments, and a clinical assessment related to psychiatric symptoms. RAs administered the assessments to study participants over one to two sessions within one week except for the clinician rated PANSS [28], which was administered separately, also within the same week, by a psychiatrist or clinical psychologist on staff at MNH or MZRH. All data were manually entered into a Research Electronic Data Capture (REDCap) [29] electronic form maintained at Duke University. Only those authors based in Tanzania had access to information that could identify individual participants during data collection (in order to facilitate follow-up) and no one had access to identifiable data after data collection was completed.

### Measures

All scales used in the study underwent a four-step process for translation and cultural validation, namely, forward-translation, back-translation, pre-testing, and finalization with expert consensus informed by previous WHO recommendations.

#### Socio-demographics

In addition to basic socio-demographics, we included the Household Hunger Scale to assess food insecurity (example item: In the past 30 days, did you or any household member go to sleep at night hungry because there was not enough food) [30]. The 6-item scale has a score from 0 to 6 with higher scores indicating higher levels of food insecurity. We used the recommended standard categorical variables indicating little to no hunger (0–1 points), moderate to severe hunger (2–3 points), and severe hunger (4–6 points).

#### Quality of life

Quality of life (QOL) for individuals with schizophrenia was measured using the brief version of the World Health Organization Quality of Life Assessment (WHOQOL_BREF). This self-report assessment has 26 questions across four domains: physical health, psychological, social relationships, and environment. Each item is rated on a five-point Likert scale ranging from 1 (not at all, very dissatisfied, very poor) to 5 (an extreme amount, very satisfied, very good); some items need reverse scoring. Higher scores indicate better QOL [31].

#### Disability

Functioning for individuals with schizophrenia was measured using the World Health Organization Disability Assessment Schedule-Second Version (WHODAS 2.0). This self-report assessment measures difficulties performing daily activities over the past 30 days. It consists of 36 Likert-formatted questions across six domains: understanding and communicating, getting around, self-care, getting along with others, life activities, and participation in society. WHO’s guidelines for the complex scoring method were used to create the total score, ranging from 0 (no disability) to 100 (full disability) [32].

#### Symptom severity

The Positive and Negative Syndrome Scale (PANSS) consists of a structured interview delivered by a clinician (study team psychiatrist or psychologist) to assess positive and negative symptoms as well as general psychopathology. The 30-items in the total PANSS score include a 7-item positive scale, 7-item negative scale, and the remaining 16 measure general psychopathology. Items are rated on a 1 (absent) to 7 (extreme) scale of increasing levels of psychopathology [28].

#### Internalized stigma

The Internalized Stigma of Mental Illness (ISMI) scale measured the individual with schizophrenia’s subjective experience of stigma. The ISMI has 29 items with a 4-point Likert scale and evaluates five areas of self-stigma: alienation, stereotype endorsement, perceived discrimination, social withdrawal, and stigma resistance [33].

#### Instrumental support

The PROMIS v2.0 (Patient-Reported Outcomes Measurement Information System), developed and validated by NIH, includes 11 items that measure whether an individual has someone who could assist with various daily tasks [34].

**Hope.** The Herth Hope Index (HHI) was used to assess hope among individuals living with schizophrenia. The HHI contains 12 items and evaluates three factors of hope: temporality and future, positive readiness and expectancy, and interconnectedness. Scores range from 12 to 48, with higher scores indicating higher levels of hopefulness [35].

#### Self-efficacy

The General Self-Efficacy Scale (GSE) assesses an individual’s belief in their ability to manage stressful situations. Respondents indicate their level of agreement with 10 items (e.g., “I can solve most problems if I invest the necessary effort”) on a Likert-type scale ranging from 1 (not true at all) to 4 (exactly true). The total score is a sum of all items and ranges between 10 and 40, with higher scores indicating more self-efficacy [36].

#### Family functioning

Individuals reported on their family’s functioning via the15-item version of the Systemic Clinical Outcome and Routine Evaluation (SCORE-15). The SCORE-15 is a questionnaire with a three-factor structure, which assesses family strengths, difficulties, and communication. Statements about family life are rated on a 5-point Likert scale from 1 (describes us: very well) to 5 (describes us: not at all). The total score ranges from 15 to 75, with a lower score indicating better family functioning. A total average score is used for analysis purposes and ranges from 1 to 5, with the same directionality as the total score [37].

#### Religiosity

The Duke University Religion Index (DUREL) is a 5-item scale that was used to measure religiosity in caregivers. The DUREL scale taps into three dimensions of religiosity: organizational religious activity, nonorganizational religious activity, and intrinsic religiosity. According to the developers of the scale, each dimension is meant to be analyzed separately. In this study, we used the Intrinsic Religiosity (IR) subscale which includes three items about a person’s degree of personal religious commitment or motivation on a 5-point scale, from 1 (definitely not true) to 5 (definitely true of me). The IR subscale score range is 3 to 15, with higher scores indicating higher religious involvement [38].

### Statistical analyses

STATA v.16 software (Stata Corp., College Station, TX) was used for all analyses. Continuous variables were summarized by their mean and standard deviation (SD). Categorical variables were summarized as counts and proportions. The two dependent variables of primary interest were patient quality of life (WHOQOL-BREF) and patient disability (WHODAS 2.0). A single item score for the WHOQOL-BREF was missing for 3/66 (4.6%) participants in the Social Relationships domain, a single item score for the WHODAS 2.0 was missing for 3 participants (Cognition domain, n = 1; Getting Along domain, n = 2) and a single item score was missing for 2/66 (3.0%) participants for the SCORE-15 Family Functioning scale. For all three instruments, a simple, single imputation of these values was performed by imputing the average of the observed responses for that same participant from all other items within the same domain. Imputed values, where applicable, were first rounded to the nearest whole integer value before complex scoring was performed. For WHOQOL, since simple scoring was used, the raw imputed value (i.e., decimal values) was included and used to generate the simple composite sum score. Complex scoring of the WHODAS 2.0 was used for all analyses.

Unstandardized Cronbach’s alpha was calculated for each psychometric instrument to assess each scale’s internal consistency (reliability) for measuring the underlying latent construct. Cronbach’s alpha was calculated on the imputed scores for WHOQOL-BREF, WHODAS and SCORE-15.

Normality of each dependent variable was assessed using histograms, quantile plots, as well performing a skewness-kurtosis test. Bivariate associations were assessed descriptively by frequencies, as well as graphically using scatter plots to assess functional relationships. Linear regression models were used to estimate correlates between independent and dependent variables. Categorical variables were fitted as disjoint indicator terms in all models. Continuous independent variables were fitted to data as both linear functions, as well as restricted cubic splines, assessed at three, four and five knot locations using Harrel’s method of placing knots at pre-determined percentiles of the distribution of the independent variable [39]. Model fit was assessed using R^2^, adjusted R^2^, and F-statistics. Model residuals were graphed to assess conditional normality.

We prioritized crude or minimally adjusted models out of a desire to maximize precision of estimated associations, and the lack of a strong heuristic model of the relationships between variables. Models treating WHODAS 2.0 score as the dependent variable were adjusted for study site (design variable in the IRGT trial), years since illness onset (binary, 4 years) and PANSS Total score (3-knot cubic spline). Models treating WHOQOL-BREF score as the dependent variable were adjusted for study site (design variable), as well as participant age (continuous linear) and PANSS Total score (3-knot cubic spline). Due to the exploratory nature of our study, inference is focused on the direction and strength of associations and not on null hypothesis significance testing [40].

### Ethics

All participants provided written informed consent for the study. Ethical approvals were received from Duke University Medical Center (Protocol No. Pro00094163), Muhimbili University of Health and Allied Sciences (MUHAS; Ref No. DA.282/298/0 I.C), Mbeya Zonal Referral Hospital (MZRH; Ref No. SZEC-2./39/R.E IV 11–13), and the Tanzanian National Institute for Medical Research (NIMR; Ref No. NIMRJHQ/R.8a/Vol. IX/3156). All procedures contributing to this work comply with the ethical standards of the relevant national and institutional committees on human experimentation and with the Helsinki Declaration of 1975, as revised in 2008.

## Results

All 66 participants completed the survey and were included in the analysis: 42 from MNH and 24 from MZRH (**Table 1**). The sample included 44 men and 22 women; the average age of participants was 33.1 years (sd = 8.2) with little difference in age by study site. Gender, years since illness onset, educational attainment, household hunger, relationship status, and recent sexual activity also did not differ substantially by study site. However, MZRH participants reported being almost exclusively Christian, whereas MNH participants reported a roughly equal distribution Christian and Muslim. Participants at MNH also reported a higher prevalence of unemployment compared with MZRH.

**Table 1:** Baseline characteristics of treatment-engaged outpatients with psychotic disorders, by study site.

	Dar es Salaam [MNH], n(%) N = 42	Mbeya [MZRH], n(%) N = 24	Total, n(%) N = 66
**Age, in years**			
Mean (SD)	31.86 (7.31)	35.13 (9.34)	33.05 (8.19)
**Age category, in years**			
≤24	9 (21.4)	1 (4.2)	10 (15.2)
25–34	15 (35.7)	12 (50.0)	27 (40.9)
35–50	18 (42.9)	11 (45.8)	29 (43.9)
**Years since illness onset**			
<4 years	16 (38.1)	7 (29.2)	23 (34.8)
≥4 years	26 (61.9)	17 (70.8)	43 (65.2)
**Gender**			
Male	26 (61.9)	18 (75.0)	44 (66.7)
Female	16 (38.1)	6 (25.0)	22 (33.3)
**Relationship Status**			
Partnered and living together	6 (14.3)	4 (16.7)	10 (15.2)
Partnered and NOT living together	6 (14.3)	6 (25.0)	12 (18.2)
Single, not in a relationship	30 (71.4)	14 (58.3)	44 (66.7)
**Sexually active in the last 3 months**			
No	31 (73.8)	22 (91.7)	53 (80.3)
Yes	11 (26.2)	2 (8.3)	13 (19.7)
**Education level**			
Primary education or less	17 (40.5)	8 (33.3)	25 (37.9)
Secondary education or higher	25 (59.5%)	16 (66.7%)	41 (62.1%)
**Religion**			
Muslim	18 (42.9)	0 (0.0)	18 (27.3)
Christian	24 (57.1)	23 (95.8)	47 (71.2)
Traditional (animist)	0 (0.0)	1 (4.2)	1 (1.5)
**Worked past 3 months**			
No	27 (64.3)	1 (4.2)	28 (42.4)
Yes	15 (35.7)	23 (95.8)	38 (57.6)
**Household Hunger**			
Little to none	34 (81.0)	22 (91.7)	56 (84.8)
Moderate or severe	8 (19.0)	2 (8.3)	10 (15.2)

The mean scores for the primary outcomes WHOQOL-BREF and WHODAS 2.0 were 76.63 and 37.50, respectively (**Table 2**). By site, participants at MNH had higher quality of life scores and lower disability, on average, than participants at MZRH. Furthermore, participants at MZRH had higher symptom severity and internalized stigma, and lower self-efficacy and hopefulness, compared with MNH participants. All of the psychometric instruments recorded good or very strong reliability (i.e., Cronbach’s alpha range of > 78% to >90%) in measuring their respective latent construct.

**Table 2:** KUPAA outpatient baseline functioning, quality of life, symptom severity, and psychosocial characteristics, by study site.

	Dar es Salaam (MNH) N = 42	Mbeya (MZRH) N = 24	Total N = 66	Cronbach’s α
**WHOQOL-BREF Raw Total Summary Score**				0.943
Mean (SD)	81.60 (19.18)	66.20 (13.20)	76.63 (18.82)	
Min, Max	45.0, 120.0	36.0, 84.0	36.0, 120.0	
**WHOQOL_BREF Domain 1: Physical Health Raw Score**				‐‐
Mean (SD)	22.98 (5.88)	19.98 (5.40)	21.89 (5.85)	
Min, Max	11.0, 34.0	11.0, 32.0	11.0, 34.0	
**WHOQOL_BREF Domain 2: Psychological Raw Score**				‐‐
Mean (SD)	20.21 (4.62)	17.54 (4.54)	19.24 (4.74)	
Min, Max	10.0, 29.0	6.0, 26.0	6.0, 29.0	
**WHOQOL_BREF Domain 3: Social Relationships Raw Score**				‐‐
Mean (SD)	8.71 (3.36)	7.04 (2.68)	8.11 (3.21)	
Min, Max	3.0, 15.0	3.0, 12.0	3.0, 15.0	
**WHOQOL_BREF Domain 4: Environnent Raw Score**				‐‐
Mean (SD)	23.93 (5.70)	20.21 (4.03)	22.58 (5.43)	
Min, Max	14.0, 36.0	12.0, 25.0	12.0, 36.0	
**WHODAS Total Summary Score_complex2**				0.955
Mean (SD)	31.86 (18.15)	47.37 (21.33)	37.50 (20.63)	
Min, Max	0.0, 72.8	9.8, 83.7	0.0, 83.7	
**WHODAS Domain 1: Cognition Summary Score complex**				‐‐
Mean (SD)	25.00 (18.21)	51.88 (29.48)	34.77 (26.20)	
Min, Max	0.0, 65.0	0.0, 95.0	0.0, 95.0	
**WHODAS Domain 2: Mobility Summary Score complex**				‐‐
Mean (SD)	22.17 (22.44)	27.34 (24.85)	24.05 (23.29)	
Min, Max	0.0, 75.0	0.0, 75.0	0.0, 75.0	
**WHODAS Domain 3: Self-care Summary Score complex**				‐‐
Mean (SD)	11.90 (14.86)	15.42 (15.03)	13.18 (14.90)	
Min, Max	0.0, 50.0	0.0, 50.0	0.0, 50.0	
**WHODAS Domain 4: Getting Along Summary Score complex**				‐‐
Mean (SD)	28.37 (22.84)	48.96 (28.37)	35.86 (26.71)	
Min, Max	0.0, 75.0	0.0, 100.0	0.0, 100.0	
**WHODAS Domain 5: Life Activities Summary Score complex**				‐‐
Mean (SD)	32.62 (21.42)	38.33 (29.44)	34.70 (24.57)	
Min, Max	0.0, 90.0	0.0, 90.0	0.0, 90.0	
**WHODAS Domain 6: Participation Summary Score complex**				‐‐
Mean (SD)	53.77 (25.78)	73.26 (18.01)	60.86 (24.97)	
Min, Max	0.0, 95.8	25.0, 100.0	0.0, 100.0	
**PANSS Total Score (Imputed)**				‐‐
Mean (SD)	40.74 (9.94)	55.04 (16.92)	45.94 (14.55)	
Min, Max	30.0, 71.0	36.0, 103.0	30.0, 103.0	
**PANSS Positive Score (Imputed)**				‐‐
Mean (SD)	9.48 (3.23)	13.75 (4.56)	11.03 (4.27)	
Min, Max	7.0, 20.0	9.0, 26.0	7.0, 26.0	
**PANSS Negative Score (Imputed)**				‐‐
Mean (SD)	9.98 (3.63)	12.96 (5.77)	11.06 (4.71)	
Min, Max	7.0, 20.0	8.0, 30.0	7.0, 30.0	
**PANSS General Score (Imputed)**				‐‐
Mean (SD)	21.29 (5.39)	28.33 (9.11)	23.85 (7.70)	
Min, Max	16.0, 35.0	18.0, 60.0	16.0, 60.0	
**Internalized Stigma** (ISMI)				0.921
Mean (SD)	2.27 (0.40)	2.51 (0.59)	2.36 (0.49)	
Min, Max	1.5, 2.8	1.2, 3.7	1.2, 3.7	
**Instrumental Support** (PROMIS)				0.957
Mean (SD)	44.47 (8.30)	34.01 (13.51)	40.67 (11.57)	
Min, Max	23.1, 55.0	11.0, 55.0	11.0, 55.0	
**Self-Efficacy** (GSE)				0.895
Mean (SD)	27.31 (6.03)	22.17 (5.20)	25.44 (6.22)	
Min, Max	11.0, 37.0	14.0, 32.0	11.0, 37.0	
**Hopefulness** (Herth Hope Index)				0.888
Mean (SD)	34.95 (7.23)	32.29 (6.30)	33.98 (6.98)	
Min, Max	20.0, 48.0	23.0, 48.0	20.0, 48.0	
**Intrinsic Religiosity** (DUREL IR)				0.778
Mean (SD)	13.19 (1.71)	12.88 (1.51)	13.08 (1.64)	
Min, Max	9.0, 15.0	9.0, 15.0	9.0, 15.0	
**Family Functioning** (SCORE-15)				0.825
Mean (SD)	2.52 (0.51)	2.84 (0.65)	2.64 (0.58)	
Min, Max	1.7, 3.9	1.9, 4.5	1.7, 4.5	

*Cronbach’s alpha values are unstandardized.

On visualization of histograms and normal-quantile plots, the two dependent variables, WHODAS 2.0 and WHOQOL-BREF, were found to be adequately normally distributed, which was further supported by Chi-square tests for skewness and kurtosis. **Table 3** shows results of regression models estimating the associations between mean WHODAS 2.0 score and participant characteristics. Crude analysis indicated a difference in mean WHODAS 2.0 score by site (mean diff: 15.52; 95% CI 5.62, 25.41); however, this difference largely went away after adjustment for PANSS Total score and age of illness onset (mean diff: 1.36; 95% CI -7.46, 10.17). Very little difference was observed in mean WHODAS 2.0 by gender on crude analysis; however, after adjustment for site and PANSS, this difference increased with higher disability associated with women. We observed large differences in WHODAS 2.0 score by years since illness onset, with participants who have been diagnosed for 4 or more years exhibiting higher disability compared with those who have been diagnosed for fewer than 4 years. This effect remained large after adjustment for site, age and PANSS Total score (mean diff: 6.57; 95% CI -2.13–15.27). Large differences in WHODAS 2.0 score by sexual activity in the past three months were observed in the crude analysis, but this almost completely disappears after adjustment.

**Table 3:** Results of univariable and multivariable linear regression, modelling disability (WHODAS 2.0) among Tanzanian outpatients with psychotic disorders, n = 66. Multivariable models are adjusted for site, years since disease onset (binary) and PANSS Total score (cubic spline), age (linear) was adjusted for only site and PANSS Total score due to collinearity with years since disease onset.

		Crude Model		Adjusted Model		
Independent variable	N	Mean WHODAS	(95% CI)	Mean WHODAS	(95% CI)	R^2^(adj.)
**Site**						**0.48**
Dar (MNH) (REF)	42	31.86	(25.89, 37.82)	37.01	(32.15, 41.87)	
Mbeya (MZRH)	24	47.37	(39.48, 55.27)	38.36	(31.67, 45.05)	
*Difference*	66	15.52	(5.62, 25.41)	1.36	(-7.46, 10.17)	
**Gender**						**0.49**
Female (REF)	22	38.09	(29.24, 46.94)	41.04	(34.62, 47.45)	
Male	44	37.20	(30.94, 43.46)	35.73	(31.24, 40.22)	
*Difference*	66	0.89	(-9.95, 11.73)	5.31	(-2.63, 13.24)	
**Years since disease onset**						**0.48**
< 4 years (REF)	23	30.53	(22.14, 38.91)	33.22	(26.46, 39.97)	
> = 4 years	43	41.23	(35.1, 47.36)	39.79	(35.02, 44.56)	
*Difference*	66	10.70	(0.31, 21.09)	6.57	(-2.13, 15.27)	
**In a relationship**						**0.48**
No (REF)	44	38.76	(32.52, 45)	38.11	(33.51, 42.70)	
Yes	22	34.98	(26.16, 43.8)	36.29	(29.66, 42.92)	
*Difference*	66	-3.78	(-14.58, 7.02)	-1.82	(-10.10, 6.46)	
**Sexually active past 3 months**						**0.47**
No (REF)	53	40.44	(34.98, 45.9)	37.78	(33.60, 41.98)	
Yes	13	25.50	(14.48, 36.52)	36.32	(27.36, 45.29)	
*Difference*	66	-14.94	(-27.24, -2.64)	-1.46	(-11.65, 8.72)	
**Education**						**0.51**
Primary or less (REF)	25	34.60	(28.22, 40.97)	42.56	(36.76, 48.35)	
Secondary or higher	41	42.26	(34.09, 50.43)	34.42	(29.90, 38.93)	
*Difference*	66	-7.66	(-18.03, 2.7)	-8.14	(-15.52, -0.76)	
**Religion**						**0.47**
Muslim (REF)	18	33.64	(23.91, 43.37)	38.48	(30.71, 46.24)	
Christian	47	38.53	(32.51, 44.55)	36.68	(32.12, 41.23)	
Traditional	1	‐‐	‐‐	‐‐	‐‐	
*Difference (Chr v*. *Muslim)*	65	4.89	(6.55, 16.34)	-1.80	(-11.22, 7.61)	
**Worked in past 3 months**						**0.47**
No (REF)	28	39.33	(32.63, 46.03)	37.45	(30.67, 44.23)	
Yes	38	35.02	(27.21, 42.82)	37.54	(31.95, 43.12)	
*Difference*	66	4.32	(-5.97, 14.6)	0.08	(-9.80, 9.96)	
**Household Hunger**						**0.51**
Little to none (REF)	56	47.83	(35, 60.66)	35.85	(31.96, 39.74)	
Moderate or severe	10	35.66	(30.23, 41.08)	46.73	(37.23, 56.22)	
*Difference*	66	12.17	(1.76, 26.1)	10.87	(0.50, 21.24)	
**Age** (continuous, linear)+	66	0.62	(0.01, 1.23)	0.31	(-0.15, 0.78)	0.47
**PANSS Total** (continuous, linear)	66	0.78	(0.49, 1.08)	0.68	(0.35, 1.01)	0.32
**PANSS Total** (cubic spline, 3 knots)	66					0.48
PANSS Total (1)		2.56	(1.75, 3.36)	2.45	(1.61, 3.30)	
PANSS Total (2)		-2.81	(-4.01, -1.6)	-2.71	(-3.93, -1.50)	
**PANSS Positive** (continuous, linear)	66	2.21	(1.13, 3.28)	1.63	(0.42, 2.85)	0.23
**PANSS Negative** (continuous, linear)	66	1.93	(0.95, 2.92)	1.57	(0.59, 2.55)	0.26
**PANSS General** (continuous, linear)	66	1.39	(0.82, 1.96)	1.18	(0.56, 1.80)	0.30
**Internalized Stigma** (continuous, linear)	66	29.15	(21.64, 36.65)	20.70	(13.94, 27.46)	0.68
**Instrumental Support** (continuous, linear)	66	-0.88	(-1.27, -0.49)	-0.60	(-0.93, -0.28)	0.57
**Self-Efficacy** (continuous, linear)	66	-2.18	(-2.8, -1.55)	-1.50	(-2.06, -0.94)	0.64
**Hopefulness** (continuous, linear)	66	-2.05	(-2.58, -1.52)	-1.39	(-1.86, -0.92)	0.67
**Intrinsic Religiosity** (continuous, linear)	66	-2.97	(-6.03, 0.08)	-0.99	(-3.40, 1.43)	0.48
**Family Functioning** (continuous, linear) [higher score is worse]	66	20.59	(13.4, 27.78)	15.93	(10.01, 21.85)	0.65

After adjustment, higher levels of reported disability (WHODAS 2.0) were found for those who had higher household food insecurity, higher internalized stigma (ISMI), lower levels of family functioning (SCORE-15), and decreasing levels of self-efficacy (GSE). A strong, positive association was observed between PANSS Total score and WHODAS 2.0. Specifically, we observed a roughly linear increase in average WHODAS score for individuals with a PANSS Total score between 30 to 55; however, results of the cubic spline regression parameters suggest that predicted WHODAS score levels off for PANSS Total values greater than about 55 (S1 File).

Results of regression models estimating the association between mean WHOQOL-BREF score and participant characteristics are found in **Table 4**. On average, WHOQOL score was observed to be higher in Dar compared Mbeya site, but this difference was significantly attenuated when adjusted for PANSS Total score and participant age. Similarly, the difference in mean WHOQOL score by years since disease onset and sexual activity in the past three months observed in the crude analysis was attenuated when adjustment was made for site, participant age, and PANSS Total score. Mean WHOQOL scores were higher (better) in participants with a secondary or higher level of education, compared to participants with a primary level of education or less, which remained after adjustment.

**Table 4:** Results of univariable and multivariable linear regression, modelling quality of life score (WHOQOL) among Tanzanian patients with psychotic disorders, n = 66. Multivariable models are adjusted for site, participant age and PANSS Total score (cubic spline).

		Crude Model		Adjusted Model		
Independent variable	N	Mean WHOQOL	(95% CI)	Mean WHOQOL	(95% CI)	R^2^(adj.)
**Site**						0.31
Dar (MNH)-(Dar) (REF)	42	81.60	(76.16, 87.03)	78.40	(73.37, 83.43)	
Mbeya (MZRH)	24	69.52	(62.33, 76.71)	75.11	(68.17, 82.06)	
*Difference*	66	-12.07	(-21.08, 3.07)	-3.29	(-12.45, 5.88)	
**Gender**						0.32
Female (REF)	22	74.22	(66.36, 82.09)	73.56	(66.92, 80.2)	
Male	44	78.69	(73.13, 84.25)	79.03	(74.38, 83.67)	
*Difference*	66	-4.47	(-14.1, 5.16)	-5.47	(-13.69, 2.75)	
**Years since disease onset**						**0.30**
< 4 years	23	81.87	(74.26, 89.48)	78.50	(71.51, 85.48)	
> = 4 years	43	74.71	(69.15, 80.27)	76.51	(71.59, 81.44)	
*Difference*	66	-7.16	(-16.58, 2.26)	-1.98	(-10.97, 7.01)	
**In a relationship**						**0.30**
No (REF)	44	77.76	(72.17, 83.35)	77.51	(72.7, 82.33)	
Yes	22	76.09	(68.18, 84)	76.59	(69.56, 83.62)	
*Difference*	66	-1.67	(-11.36, 8.01)	-0.93	(-9.79, 7.94)	
**Sexually active past 3 months**						**0.30**
No (REF)	53	75.67	(70.64, 80.7)	77.15	(72.79, 81.51)	
Yes	13	83.46	(73.31, 93.61)	77.42	(67.92, 86.93)	
*Difference*	66	7.79	(-3.53, 19.12)	0.27	(-10.58, 11.12)	
**Education**						**0.36**
Primary or less (REF)	25	70.64	(63.51, 77.77)	70.98	(64.59, 77.37)	
Secondary or higher	41	81.21	(75.64, 86.77)	81.00	(76.15, 85.85)	
Difference	66	10.57	(1.52, 19.61)	10.03	(1.57, 18.48)	
**Religion**						**0.30**
Muslim (REF)	18	80.28	(71.52, 89.04)	76.51	(68.48, 84.53)	
Christian	47	76.22	(70.8, 81.65)	77.67	(72.96, 82.38)	
Traditional	1	‐‐	‐‐	‐‐	‐‐	
*Difference (Chris v*. *Muslim)*	65	-4.05	(-14.36, 6.25)	1.16	(-8.56, 10.89)	
**Worked in the past 3 months**						**0.30**
No (REF)	28	79.36	(72.38, 86.34)	76.97	(69.99, 83.95)	
Yes	38	75.62	(69.63, 81.61)	77.38	(71.63, 83.13)	
*Difference*	66	-3.74	(-12.94, 5.46)	0.41	(-9.76, 10.58)	
**Household Hunger**						**0.31**
Little or none (REF)	56	78.51	(73.62, 83.4)	78.10	(73.98, 82.22)	
Moderate or severe	10	69.90	(58.33, 81.47)	72.18	(62.13, 82.23)	
*Difference*	66	-8.61	(-21.17, 3.95)	-5.92	(-16.9, 5.06)	
**Age** (continuous, linear)	66	-0.67	(-1.2, -0.13)	-0.45	(-0.93, 0.02)	0.31
**PANSS Total** (continuous, linear)	66	-0.42	(-0.72, -0.12)	-0.27	(-0.6, 0.06)	0.15
**PANSS Total** (cubic spline, 3 knots)	66					0.31
PANSS Total (1)		-2.08	(-2.91, -1.24)	-1.86	(-2.74, -0.99)	
PANSS Total (2)		2.62	(1.38, 3.88)	2.43	(1.17, 3.68)	
**PANSS Positive** (continuous, linear)	66	-1.12	(-2.17, -0.08)	-0.60	(-1.74, 0.54)	0.13
**PANSS Negative** (continuous, linear)	66	-0.58	(-1.55, 0.38)	-0.14	(-1.1, 0.82)	0.12
**PANSS General** (continuous, linear)	66	-0.93	(-1.48, -0.38)	-0.70	(-1.29, -0.1)	0.19
**Internalized Stigma** (continuous, linear)	66	-28.82	(-34.78, -22.85)	-23.78	(-30.4, -17.17)	0.62
**Instrumental Support** (continuous, linear)	66	0.79	(0.44, 1.13)	0.61	(0.28, 0.95)	0.43
**Self-Efficacy** (continuous, linear)	66	1.95	(1.39, 2.51)	1.57	(1, 2.14)	0.53
**Hopefulness** (continuous, linear)	66	2.17	(1.8, 2.55)	1.87	(1.49, 2.26)	0.73
**Intrinsic Religiosity** (continuous, linear)	66	2.63	(-0.1, 5.37)	1.58	(-0.8, 3.96)	0.32
**Family Functioning** (1–5, impute, continuous, linear; higher score is worse)	66	-18.71	(-25.08, -12.34)	-14.91	(-21.13, -8.69)	0.49

Similar to the WHODAS 2.0, mean WHOQOL-BREF scores were approximately linearly associated with most mental health measures. Specifically, higher levels of self-efficacy (GSE), instrumental support (PROMIS), and hopefulness (HHI) were associated with better quality of life, higher WHOQOL scores. However, higher levels of internalized stigma (ISMI) and worse family functioning (higher scores) were associated with lower mean WHOQOL-BREF scores indicating lower quality of life. Cubic spline regression suggests a negative linear association between WHOQOL-BREF and PANSS Total for PANSS Total values up to approximately 55, at which point the relationship inverses; although the lack of precision in this estimated relationship, especially for high values of PANSS where we had few observations, makes interpretation of this relationship difficult (see S1 File). For all analyses, a complete-case analysis (excluding missing item scores) did not meaningfully change the study results.

## Discussion

Our study describes a Tanzanian treatment-engaged population of adults with schizophrenia and estimates correlates of quality of life and disability in this population. Disability and quality of life score comparisons across countries, even within sub-Saharan Africa, are difficult but interpretation of the levels should include attention to the fact that our study population was currently in treatment, indicating some degree of functioning, and relatedly, perhaps a higher self-reported quality of life compared to untreated populations. Furthermore, despite the fact that we focused our study on a care-seeking population, we observed a relatively high variability in disability scores (coefficient of variation of WHODAS 2.0 = 0.56).

Our study populations were also quite different by study site, with individuals attending outpatient psychiatric services in Mbeya exhibiting higher symptom severity compared with those in Dar es Salaam. This might be due, in part, to the difference in duration of illness, with a higher proportion of Mbeya participants living with a diagnosis for at least four years. Differences in other psychosocial measures by site largely disappeared after controlling for symptom severity.

Similar to previous studies, we found the lowest scores for WHOQOL-BREF to be in the *social relationships* domain, suggesting that interventions that are able to target socialization may have the strongest impact for improving quality of life scores. Similarly, the lowest domain scores for the WHODAS 2.0 were in *getting along*. Despite this, the *getting along* domain measures how well a person is able to interact with other people, another important social component. This domain reflects not just an individual’s skills but also how the social environment interacts with them (e.g. interactions with strangers, friendships, romantic relationships). Individual skills building will not overcome some of these issues if there is substantial familial and community-based stigma.

Our study found scant evidence that average quality of life scores differed by sociodemographic factors, the exception being that participants with higher education were found to have higher quality of life, even when comparing participants with similar symptom severity. The level of education may be important in quality of life on its own, or education might be confounding a causal association, such as employment or hunger/poverty. We did observe lower quality of life and higher disability for those participants with moderate or severe household hunger; however, our estimates were imprecise. Results estimating associations between disability and sociodemographic factors were largely consistent with results observed for quality of life. Specifically, we found little evidence that disability scores differed by most sociodemographic factors, with the exception of education level.

For both quality of life and disability, we observed strong linear associations with several psychosocial measures, even after adjustment for symptom severity. Study participants with higher internalized stigma and lower family functioning reported, on average, lower quality of life, and participants with higher instrumental support, self-efficacy and hopefulness, reported higher quality of life scores. A very similar pattern was observed for the relationship between psychosocial measures and participants’ disability. Both sets of findings add to the literature [27] suggesting that individuals living with schizophrenia who feel supported, hopeful and who have a sense of self-efficacy, may also be better off in terms of higher quality of life and lower disability. This has implications for developing recovery-oriented psychosocial treatment services for this population. If these factors mediate the effects of treatment on quality of life and disability, then we should develop treatment programs that directly impact these factors. How hopefulness and self-efficacy may work as mechanisms of action for our outcomes are still being explored in the wider literature but an important component of hope and self-efficacy is having goals and taking actions, both of which could influence specific items on measures of disability and quality of life [41, 42].

Our study is not without limitations. Participants for this study included only adults ages 18–50 who were actively attending outpatient psychiatric treatment at the time of enrollment; thus, inference from our study should be limited to a care-seeking population and may not be generalizable to all Tanzanian persons with schizophrenia. In addition, our study population did not have their psychiatric diagnoses confirmed by a standardized measure (e.g. SCID or MINI). We also note that we did not include medication adherence and physical health co-morbidities as potential confounders due to some measurement issues. Given our study sites were located in urban areas, our sample is also more likely to over-represent those with more education. As with all psychometric evaluations, our psychosocial measures are subject to measurement error, which could lead to bias in both estimates of prevalence and measures of association (i.e., mean difference). While this should cause our measures of association to usually be biased in the direction of the null, and therefore, conservative, this is not guaranteed. We also note that the PANSS, as a clinician-administered instrument, may have varied due to clinical judgement and we do not have a measure of inter-rater reliability. Furthermore, regression modeling was exploratory and multivariable models were not parameterized based on theory. For this reason, hypothesis tests were not performed and inference focused on the direction and size of the effects. And while we limited the variable adjustment set, we cannot rule out the possibility that adjustment for one or more variables could have led to collider stratification bias. Regression coefficients should be interpreted as adjusted total direct effects, conditional on other variables in the model.

With global calls for enhancing treatment of psychoses in low-resource settings, low- and middle-income countries like Tanzania are re-evaluating their psychiatric services and discussing ways forward that include more psychosocial programming in tandem with medication management which has historically been the focus. Country level evidence on factors associated with disability and quality of life, can help clinicians and policymakers, as well as consumers of mental health services, better understand the opportunities to improve quality of life and reduce disability offered by targeted and strategic psychosocial services and family and community education.

## Supporting information

S1 File Supplemental figures. (DOCX)

S1 Checklist STROBE checklist. (DOCX)
