# Supplementary material for: Functioning and quality of life among treatment-engaged adults with psychotic disorders in urban Tanzania: Baseline results from the KUPAA clinical trial
Source: PLoS One. 2024 Jun 18;19(6):e0304367. doi: 10.1371/journal.pone.0304367 (PMC11185462; doi:10.1371/journal.pone.0304367)
Supplement: S1 File — (DOCX) [file pone.0304367.s001.docx]

**Supplemental File 1.**

Figures: Relationship between predicted WHODAS 2.0 and PANSS Total score, adjusted for study site and years since illness onset (top panel); relationship between predicted WHOQOL-BREF and PANSS Total score, adjusted for site and age (bottom panel). For both models, PANSS Total Score is modelled as a 3-knot cubic spline.
