# Supplementary material for: Functioning and quality of life among treatment-engaged adults with psychotic disorders in urban Tanzania: Baseline results from the KUPAA clinical trial
Source: PLoS One. 2024 Jun 18;19(6):e0304367. doi: 10.1371/journal.pone.0304367 (PMC11185462; doi:10.1371/journal.pone.0304367)
Supplement: S1 Checklist — (DOCX) [file pone.0304367.s002.docx]

STROBE Statement—checklist of items that should be included in reports of observational studies

|  | Item No. | Recommendation | Page  No. | Relevant text from manuscript |
| --- | --- | --- | --- | --- |
| **Title and abstract** | 1 | (*a*) Indicate the study’s design with a commonly used term in the title or the abstract | 2 | This study analyzed cross-sectional data from 66 individuals enrolled in the Kuwezeshana… |
|  |  | (*b*) Provide in the abstract an informative and balanced summary of what was done and what was found | 2 | Adjusted analyses indicated that.. less self-stigma, and better family functioning. |
| Introduction | | | |  |
| Background/rationale | 2 | Explain the scientific background and rationale for the investigation being reported | 4 | The implementation and evaluation of culturally adapted, evidence-based psychosocial interventions aimed at addressing these factors, such as Family Psychoeducation (5,19) could improve functioning and disability… |
| Objectives | 3 | State specific objectives, including any prespecified hypotheses | 5 | The primary aim of the study was to explore the associations… We hypothesize that perceived level of support |
| Methods | | | |  |
| Study design | 4 | Present key elements of study design early in the paper | 5 | This current study uses cross-sectional, pre-intervention baseline data from an individually randomized group treatment (IRGT) trial in two tertiary level hospital facilities in Dar es Salaam and Mbeya, Tanzania. |
| Setting | 5 | Describe the setting, locations, and relevant dates, including periods of recruitment, exposure, follow-up, and data collection | 6-8 | Baseline data collection occurred between September 3, 2019 and November 1, 2019… |
| Participants | 6 | (*a*) *Cohort study*—Give the eligibility criteria, and the sources and methods of selection of participants. Describe methods of follow-up  *Case-control study*—Give the eligibility criteria, and the sources and methods of case ascertainment and control selection. Give the rationale for the choice of cases and controls  *Cross-sectional study*—Give the eligibility criteria, and the sources and methods of selection of participants | 7 | Adults attending one of the two study psychiatric outpatient clinics were eligible for inclusion if… rendered the individual unable to provide informed consent. |
|  |  | (*b*) *Cohort study*—For matched studies, give matching criteria and number of exposed and unexposed  *Case-control study*—For matched studies, give matching criteria and the number of controls per case |  |  |
| Variables | 7 | Clearly define all outcomes, exposures, predictors, potential confounders, and effect modifiers. Give diagnostic criteria, if applicable | 7-8 | Adults attending one of the two study psychiatric outpatient clinics… psychiatrists at each study site were available to assess and determine capacity to consent to research participation. |
| Data sources/ measurement | 8* | For each variable of interest, give sources of data and details of methods of assessment (measurement). Describe comparability of assessment methods if there is more than one group | 8-11 | All scales used in the study underwent a four-step process for translation and cultural validation… The IR subscale score range is 3 to 15, with higher scores indicating higher religious involvement. |
| Bias | 9 | Describe any efforts to address potential sources of bias | 7 | If RAs had any concerns about capacity, psychiatrists at each study site were available to assess and determine capacity to consent to research participation. |
| Study size | 10 | Explain how the study size was arrived at | 6 | However, we estimate that our sample of 66 patient participants will provide 82% power to detect a correlation coefficient between two continuous variables |

| Quantitative variables | 11 | Explain how quantitative variables were handled in the analyses. If applicable, describe which groupings were chosen and why | 12-13 | Normality of each dependent variable… Due to the exploratory nature of our study, inference is focused on the direction and strength of associations and not on precision of estimates. |
| --- | --- | --- | --- | --- |
| Statistical methods | 12 | (*a*) Describe all statistical methods, including those used to control for confounding | 11-13 | STATA v.16 software (Stata Corp., College Station, TX) was used for all analyses…. Due to the exploratory nature of our study, inference is focused on the direction and strength of associations and not on precision of estimates. |
|  |  | (*b*) Describe any methods used to examine subgroups and interactions | N/A |  |
|  |  | (*c*) Explain how missing data were addressed | 12 | A single item score for the WHOQOL-BREF was missing for 3/66 (4.6%) participants in the Social Relationships domain, a single item score for the WHODAS 2.0 was missing for 3 participants (Cognition domain, n=1; Getting Along domain, n=2) and a single item score was missing for 2/66 (3.0%) participants for the SCORE-15 Family Functioning scale. For all three instruments, a simple, single imputation of these values was performed … |
|  |  | (*d*) *Cohort study*—If applicable, explain how loss to follow-up was addressed  *Case-control study*—If applicable, explain how matching of cases and controls was addressed  *Cross-sectional study*—If applicable, describe analytical methods taking account of sampling strategy | 13 | We prioritized crude or minimally adjusted models out of a desire to maximize precision of estimated associations, |
|  |  | (*e*) Describe any sensitivity analyses | N/A |  |
| Results | | | | |
| Participants | 13* | (a) Report numbers of individuals at each stage of study—eg numbers potentially eligible, examined for eligibility, confirmed eligible, included in the study, completing follow-up, and analysed | 14 | All 66 participants completed the survey… |
|  |  | (b) Give reasons for non-participation at each stage |  |  |
|  |  | (c) Consider use of a flow diagram | N/A |  |
| Descriptive data | 14* | (a) Give characteristics of study participants (eg demographic, clinical, social) and information on exposures and potential confounders | 14 | All 66 participants completed the survey… Participants at MNH also reported a higher prevalence of unemployment compared with MZRH. |
|  |  | (b) Indicate number of participants with missing data for each variable of interest | 11-12 | A single item score for the WHOQOL-BREF was missing for 3/66 (4.6%) participants in the Social Relationships domain, a single item score for the WHODAS 2.0 was missing for 3 participants (Cognition domain, n=1; Getting Along domain, n=2) and a single item score was missing for 2/66 (3.0%) participants for the SCORE-15 Family Functioning scale. |
|  |  | (c) *Cohort study*—Summarise follow-up time (eg, average and total amount) |  |  |
| Outcome data | 15* | *Cohort study*—Report numbers of outcome events or summary measures over time |  |  |
|  |  | *Case-control study—*Report numbers in each exposure category, or summary measures of exposure |  |  |
|  |  | *Cross-sectional study—*Report numbers of outcome events or summary measures | 15-22 | The mean scores for the primary outcomes WHOQOL-BREF and WHODAS 2.0… (also Tables) |
| Main results | 16 | (*a*) Give unadjusted estimates and, if applicable, confounder-adjusted estimates and their precision (eg, 95% confidence interval). Make clear which confounders were adjusted for and why they were included | 14-22 | Tables |
|  |  | (*b*) Report category boundaries when continuous variables were categorized | N/A |  |
|  |  | (*c*) If relevant, consider translating estimates of relative risk into absolute risk for a meaningful time period | N/A |  |

| Other analyses | 17 | Report other analyses done—eg analyses of subgroups and interactions, and sensitivity analyses | N/A |  |
| --- | --- | --- | --- | --- |
| Discussion | | | | |
| Key results | 18 | Summarise key results with reference to study objectives | 22-24 | Our study describes a Tanzanian treatment-engaged population of adults with schizophrenia and estimates correlates of quality of life and disability in this population… |
| Limitations | 19 | Discuss limitations of the study, taking into account sources of potential bias or imprecision. Discuss both direction and magnitude of any potential bias | 24-25 | Our study is not without limitations… we cannot rule out the possibility that adjustment for one or more variables could have led to collider stratification bias. |
| Interpretation | 20 | Give a cautious overall interpretation of results considering objectives, limitations, multiplicity of analyses, results from similar studies, and other relevant evidence | 23-25 | Similar to previous studies, we found the lowest scores for WHOQOL-BREF to be in the social relationships domain… |
| Generalisability | 21 | Discuss the generalisability (external validity) of the study results | 24-25 | Participants for this study included only adults ages 18-50 who were actively attending outpatient psychiatric treatment at the time of enrollment; thus, inference from our study should be limited to a care-seeking population, and may not be generalizable to all Tanzanian persons with schizophrenia. |
| Other information | |  | | |
| Funding | 22 | Give the source of funding and the role of the funders for the present study and, if applicable, for the original study on which the present article is based | 26 | The study was funded by The National Institute of Mental Health (NIMH) R34MH106663. |

*Give information separately for cases and controls in case-control studies and, if applicable, for exposed and unexposed groups in cohort and cross-sectional studies.

**Note:** An Explanation and Elaboration article discusses each checklist item and gives methodological background and published examples of transparent reporting. The STROBE checklist is best used in conjunction with this article (freely available on the Web sites of PLoS Medicine at http://www.plosmedicine.org/, Annals of Internal Medicine at http://www.annals.org/, and Epidemiology at http://www.epidem.com/). Information on the STROBE Initiative is available at www.strobe-statement.org.
